# Supplementary material for: An ensemble based approach using a combination of clustering and classification algorithms to enhance customer churn prediction in telecom industry
Source: PeerJ Comput Sci. 2022 Feb 22;8:e854. doi: 10.7717/peerj-cs.854 (PMC9044233; doi:10.7717/peerj-cs.854)
Supplement: Supplemental Information 1 [file peerj-cs-08-854-s001.docx]

Table 1: Map clustering on label

| **Id** | **Cluster** | **Class** | **Prediction(Class)** |
| --- | --- | --- | --- |
| 1 | Cluster_0 | 0 | 0 |
| 2 | Cluster_1 | 0 | 0 |
| 3 | Cluster_0 | 0 | 0 |
| 4 | Cluster_0 | 0 | 1 |
| 5 | Cluster_0 | 1 | 0 |
| 6 | Cluster_1 | 0 | 0 |
| 7 | Cluster_1 | 0 | 1 |
| 8 | Cluster_1 | 1 | 0 |
| 9 | Cluster_1 | 1 | 0 |
| 10 | Cluster_1 | 1 | 1 |
